# Supplementary material for: Short linear motifs in intrinsically disordered regions modulate HOG signaling capacity
Source: BMC Syst Biol. 2018 Jul 3;12:75. doi: 10.1186/s12918-018-0597-3 (PMC6029073; doi:10.1186/s12918-018-0597-3)
Supplement: Supplementary file 1 — Table S1. Predicted short linear motifs (pSLiMs) in the hypotonic stress and canonical Wnt signaling pathways. A. Signaling components of the hypotonic stress pathway in S.cerevisiae identified from KEGG, with 36 SLiMs predicted in 11 hypotonic stress signaling proteins using PhyloHMM. B. Signaling components of the human canonical Wnt signaling pathway identified from KEGG, with 24 SLiMs predicted in 16 Wnt signaling proteins using SLiMPrints. Periods indicate positions where any amino acid can match. (PDF 32 kb) [file 12918_2018_597_MOESM1_ESM.pdf]

Table S1: Predicted short linear motifs (pSLIMs) in the Hypotonic stress and canonical Wnt signaling pathways.

A. Signaling components of the Hypotonic stress pathway in *S.cerevisiae* identified from KEGG, with 36 SLiMs predicted in 11 Hypotonic stress signaling proteins using PhyloHMM.

gene_ID	Uniprot_ID	gene_symbol	full name	pSLiM_ID	pSLiM
1	P36027	Mid2	Cell wall integrity sensor MID2	1	SPLG
2	P53832	Wsc2	Cell wall integrity and stress response component 2	2	TRT
2	P53832	Wsc2	Cell wall integrity and stress response component 2	3	GAIAGVV
2	P53832	Wsc2	Cell wall integrity and stress response component 2	4	RF
2	P53832	Wsc2	Cell wall integrity and stress response component 2	5	SLPD
2	P53832	Wsc2	Cell wall integrity and stress response component 2	6	IVNPDN
3	P38631	Fks1	1,3 beta-glucan synthase component FKS1	7	YGQP
3	P38631	Fks1	1,3 beta-glucan synthase component FKS1	8	YYDPN
3	P38631	Fks1	1,3 beta-glucan synthase component FKS1	9	NDTG
3	P38631	Fks1	1,3 beta-glucan synthase component FKS1	10	TWSTI
4	P24583	Pkc1	Protein kinase C-like 1	11	FT
4	P24583	Pkc1	Protein kinase C-like 1	12	TLDP
4	P24583	Pkc1	Protein kinase C-like 1	13	SPQK
5	Q01389	Bck1	Serine/threonine-protein kinase BCK1/SLK1/SSP31	14	SS
5	Q01389	Bck1	Serine/threonine-protein kinase BCK1/SLK1/SSP31	15	ST
5	Q01389	Bck1	Serine/threonine-protein kinase BCK1/SLK1/SSP31	16	RSTSES
5	Q01389	Bck1	Serine/threonine-protein kinase BCK1/SLK1/SSP31	17	IP
5	Q01389	Bck1	Serine/threonine-protein kinase BCK1/SLK1/SSP31	18	KFKR
5	Q01389	Bck1	Serine/threonine-protein kinase BCK1/SLK1/SSP31	19	YPQTPS
5	Q01389	Bck1	Serine/threonine-protein kinase BCK1/SLK1/SSP31	20	NYWN
5	Q01389	Bck1	Serine/threonine-protein kinase BCK1/SLK1/SSP31	21	RSNS
5	Q01389	Bck1	Serine/threonine-protein kinase BCK1/SLK1/SSP31	22	SSDDIIWS
6	P32490	Mkk1	MAP kinase kinase MKK1/SSP32	23	GSS
6	P32490	Mkk1	MAP kinase kinase MKK1/SSP32	24	RPAPPS
6	P32490	Mkk1	MAP kinase kinase MKK1/SSP32	25	VDQL
7	P32491	Mkk2	MAP kinase kinase MKK2/SSP33	26	SS
7	P32491	Mkk2	MAP kinase kinase MKK2/SSP33	27	RPVPPP
8	P40457	Mlp2	Protein MLP2	28	PLS
8	P40457	Mlp2	Protein MLP2	29	LE
9	Q12224	Rlm1	Transcription factor RLM1	30	SSSSS
10	P25302	Swi4	Regulatory protein SWI4	31	NPSPLQNLVFQTPQQ
10	P25302	Swi4	Regulatory protein SWI4	32	YSATQKPLQFF
10	P25302	Swi4	Regulatory protein SWI4	33	GSS
11	P09959	Swi6	Regulatory protein SWI6	34	KR
11	P09959	Swi6	Regulatory protein SWI6	35	LGSPLKK
11	P09959	Swi6	Regulatory protein SWI6	36	HDL

B. Signaling components of the human canonical Wnt signaling pathway identified from KEGG, with 24 SLiMs predicted in 16 Wnt signaling proteins using SLiMPrints. Periods indicate positions where any amino acid can match.

gene_ID	Uniprot_ID	gene_symbol	full name	pSLiM_ID	pSLiM
1	O15169	AXIN1	Axin-1	1	KK
1	O15169	AXIN1	Axin-1	2	L..HV.RV
2	Q9Y2T1	AXIN2	Axin-2	3	LP
3	P35222	CTNNB1	Catenin beta-1	4	R.R.A.FP
3	P35222	CTNNB1	Catenin beta-1	5	W.DTDL
4	O14640	DVL1	Segment polarity protein dishevelled homolog DVL1	6	G..S..S
4	O14640	DVL1	Segment polarity protein dishevelled homolog DVL1	7	S..GS
5	O14641	DVL2	Segment polarity protein dishevelled homolog DVL2	8	S..SF..A.G
5	O14641	DVL2	Segment polarity protein dishevelled homolog DVL2	9	G..RR
6	Q92997	DVL3	Segment polarity protein dishevelled homolog DVL3	10	S..SG
7	Q9UJU2	LEF1	Lymphoid enhancer-binding factor 1	11	I.HPA
7	Q9UJU2	LEF1	Lymphoid enhancer-binding factor 1	12	R.PYP
8	O75197	LRP5	Low-density lipoprotein receptor-related protein 5	13	DR.H..G.S
9	O43318	MAP3K7	Mitogen-activated protein kinase kinase kinase 7	14	LD..LQP
10	Q969G9	NKD1	Protein naked cuticle homolog 1	15	KHK.R.K
11	Q969F2	NKD2	Protein naked cuticle homolog 2	16	P..QRHE
12	Q9HC62	SENP2	Sentrin-specific protease 2	17	RR
13	Q13485	SMAD4	Mothers against decapentaplegic homolog 4	18	W.G..T..YT
13	Q13485	SMAD4	Mothers against decapentaplegic homolog 4	19	TIQH.P
14	Q9HCS4	TCF7L1	Transcription factor 7-like 1	20	KK.C..Y
14	Q9HCS4	TCF7L1	Transcription factor 7-like 1	21	SP
15	Q9NQB0	TCF7L2	Transcription factor 7-like 2	22	E.L
