# Supplementary material for: Short linear motifs in intrinsically disordered regions modulate HOG signaling capacity
Source: BMC Syst Biol. 2018 Jul 3;12:75. doi: 10.1186/s12918-018-0597-3 (PMC6029073; doi:10.1186/s12918-018-0597-3)
Supplement: Supplementary file 2 — Table S2. P-values from Wilcoxon Rank Sum Tests comparing the distribution of KL divergence from mutants to wild-type pools. (PDF 37 kb) [file 12918_2018_597_MOESM2_ESM.pdf]

Table S2

| strain_ID | mutation | status | P-value KL-divergence difference from wt | multiple testing correction (17 tests) | Median of KL-divergence - between mutant and wild-type | Median of KL-divergence - between mutant and model | Replicates (n) |
|---|---|---|---|---|---|---|---|
| YBS117 | Opy2 Δ233-45 | known | 5.72E-05 | 0.001 | 0.070 | 0.064 | 6 |
| YBS146 | Ste20 Δ469-80 | known | 0.955 | 1 | 0.013 | 0.060 | 5 |
| YBS105 | Hot1 Δ373-85 | known | 5.85E-07 | 9.95E-06 | 1.526 | 0.153 | 11 |
| YBS92 | Pbs2 Δ91-100 | known | n/d | n/d | | | |
| YBS91 | Wild-type | na | | | 0.012 | 0.053 | 38 |
| YBS95 | Sho1 Δ217-31 | novel | 0.023 | 0.394 | 0.018 | 0.055 | 7 |
| YBS144 | Ste20 Δ66-71 | novel | 0.925 | 1 | 0.018 | 0.108 | 5 |
| YBS138 | Pbs2 Δ249-55 | novel | 0.532 | 1 | 0.014 | 0.080 | 5 |
| YBS140 | Pbs2 Δ281-5 | novel | 0.116 | 1 | 0.038 | 0.091 | 5 |
| YBS142 | Pbs2 Δ292-8 | novel | 0.191 | 1 | 0.014 | 0.105 | 5 |
| YBS106 | Nup133 Δ8-13 | novel | 0.085 | 1 | -0.650 | 0.388 | 8 |
| YBS94 | Ste50 Δ341-6 | novel | 0.001 | 0.016 | 0.047 | 0.058 | 7 |
| YBS121 | Ste11 Δ379-82 | novel | 2.51E-03 | 0.043 | 0.125 | 0.080 | 7 |
| YBS118 | Pbs2 Δ180-8 | novel | 3.79E-04 | 0.006 | 0.121 | 0.104 | 6 |
| YBS119 | Pbs2 Δ269-74 | novel | 3.33E-05 | 0.001 | 2.778 | 0.182 | 7 |
| YBS120 | Pbs2 Δ661-8 | novel | 1.19E-03 | 0.020 | 0.050 | 0.046 | 7 |
| YBS108 | Sin3 Δ541-8 | novel | 0.002 | 0.042 | 0.054 | 0.036 | 8 |
| YBS148 | Sch9 Δ178-89 | novel | 1.04E-04 | 0.002 | 0.269 | 0.099 | 6 |
