# Supplementary figures and images for: Short linear motifs in intrinsically disordered regions modulate HOG signaling capacity
Source: BMC Syst Biol. 2018 Jul 3;12:75. doi: 10.1186/s12918-018-0597-3 (PMC6029073; doi:10.1186/s12918-018-0597-3)

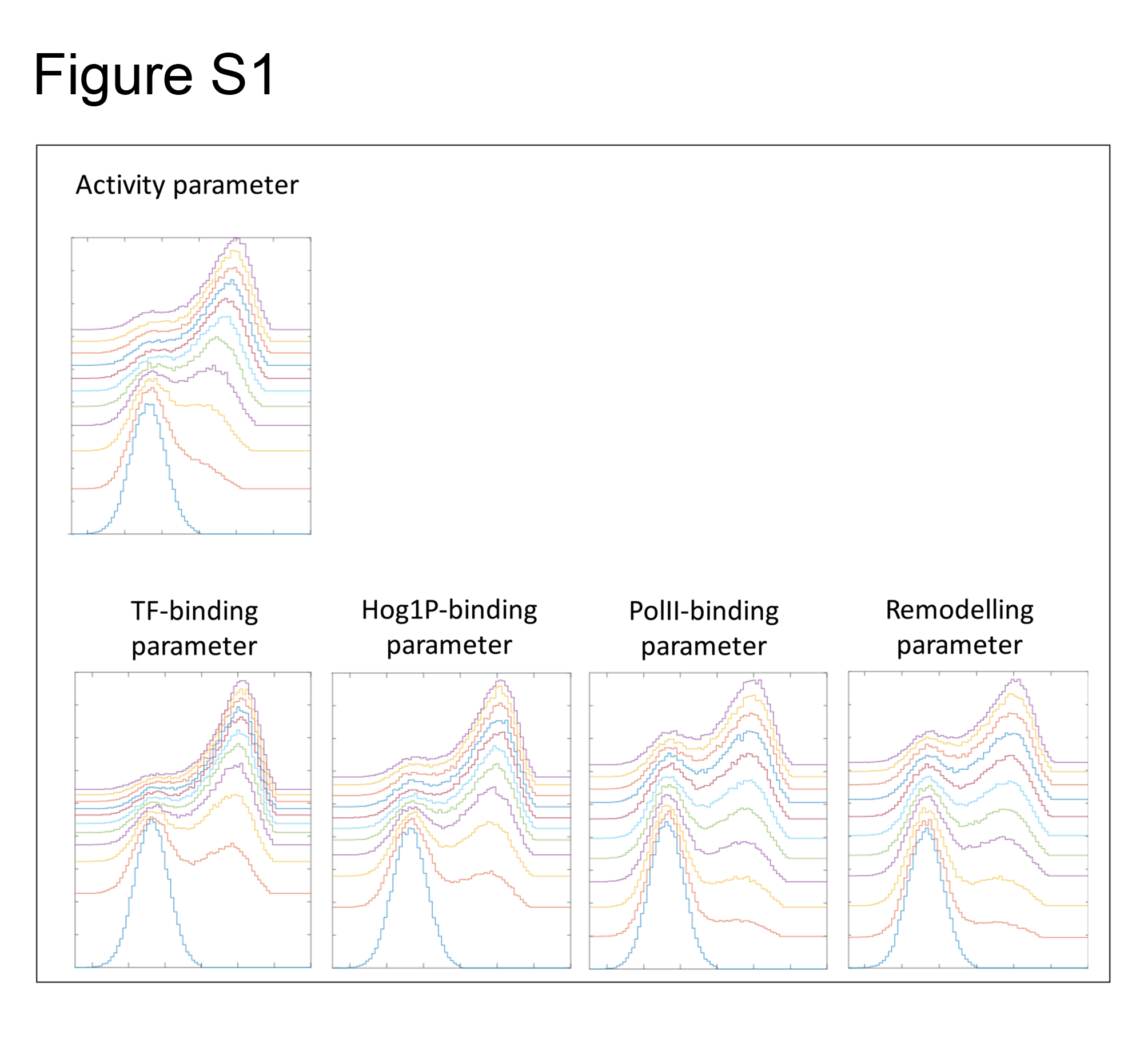

Supplement: Supplementary file 3 — : Figure S1. Effects of perturbations in model parameters fall into two groups. The activity parameter that affects the pathway activation steps upstream of the MAPK Hog1 (referred to as c_active in [21], top panel) affects both the proportion of cells that activate the pathway (right mode of distribution) as well as the maximum activity of the pathway (location of the right mode), such that as the parameter is reduced, fewer cells activate the pathway and those that do, do so to a lesser extent. The bottom panel shows that the four parameters that affect the slow transcriptional steps below Hog1 (referred to as c_tfon, c_hogon, c_remon, c_polon in [21]) affect the proportion of cells in the right mode, but not the location of the mode, so the distributions appear more bimodal. Each plot contains predictions of the model where the original parameter value has been permuted in the range from 0 to 50% of the original parameter value in increments of 5%. (TIF 736 kb) [file 12918_2018_597_MOESM3_ESM.tif]

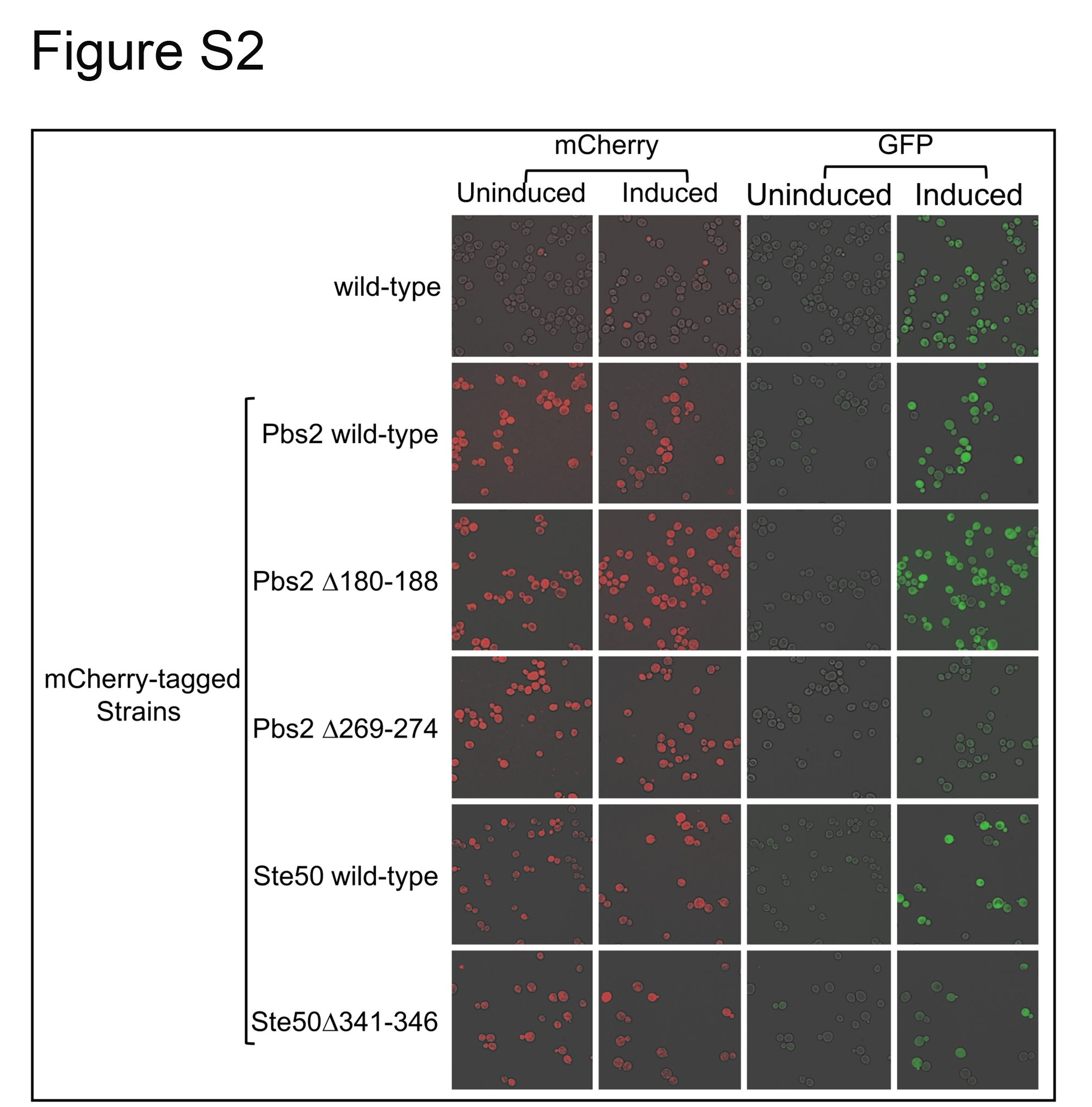

Supplement: Supplementary file 4 — : Figure S2. Controls for mutant protein expression. Confocal images of dual fluorescent reporter strains with a pSTL1::GFP HOG pathway reporter combined with C-terminal mCherry fusions of Pbs2 Δ180–188, Pbs2 Δ269–274, Ste50 Δ341–346, wild-type Pbs2 and wild-type Ste50 before and after a 60 min induction with 0.4 M NaCl. The top row shows the pSTL1::GFP wild-type strain (no mCherry) for comparison. The left two columns show the mCherry channel, and we find no difference of mCherry fusion protein expression levels or localization in SLiM deletion strains as compared to their wild-type counterparts. The right two columns show the GFP channel and confirm that the HOG pathway has been induced as expected (based on our flow cytometry results) in the mCherry tagged strains. (TIF 1835 kb) [file 12918_2018_597_MOESM4_ESM.tif]
